# Optimising complementary soft tissue synchrotron X-ray microtomography for reversibly-stained central nervous system samples

**DOI:** 10.1038/s41598-018-30520-8

**Published:** 2018-08-13

**Authors:** Merrick C. Strotton, Andrew J. Bodey, Kazimir Wanelik, Michele C. Darrow, Esau Medina, Carl Hobbs, Christoph Rau, Elizabeth J. Bradbury

**Affiliations:** 1 King’s College London, Wolfson Centre for Age Related Diseases, Institute of Psychiatry, Psychology & Neuroscience, Guy’s Campus, London Bridge, London, SE1 1UL UK; 2 Diamond-Manchester Imaging Branchline I13-2, Diamond Light Source, Oxfordshire, OX11 0DE UK; 3 Beamline B24, Diamond Light Source, Oxfordshire, OX11 0DE UK

## Abstract

Synchrotron radiation microtomography (SRμCT) is a nominally non-destructive 3D imaging technique which can visualise the internal structures of whole soft tissues. As a multi-stage technique, the cumulative benefits of optimising sample preparation, scanning parameters and signal processing can improve SRμCT imaging efficiency, image quality, accuracy and ultimately, data utility. By evaluating different sample preparations (embedding media, tissue stains), imaging (projection number, propagation distance) and reconstruction (artefact correction, phase retrieval) parameters, a novel methodology (combining reversible iodine stain, wax embedding and inline phase contrast) was optimised for fast (~12 minutes), high-resolution (3.2–4.8 μm diameter capillaries resolved) imaging of the full diameter of a 3.5 mm length of rat spinal cord. White-grey matter macro-features and micro-features such as motoneurons and capillary-level vasculature could then be completely segmented from the imaged volume for analysis through the shallow machine learning SuRVoS Workbench. Imaged spinal cord tissue was preserved for subsequent histology, establishing a complementary SRμCT methodology that can be applied to study spinal cord pathologies or other nervous system tissues such as ganglia, nerves and brain. Further, our ‘single-scan iterative downsampling’ approach and side-by-side comparisons of mounting options, sample stains and phase contrast parameters should inform efficient, effective future soft tissue SRμCT experiment design.

## Introduction

The central nervous system (CNS) is an organ with 3D spatial organisation that is essential to its proper function. In the spinal cord, this is exemplified by the lamina distribution of cell bodies through the grey matter and spatial segregation of ascending and descending white matter tracts. Amongst this are a branching vasculature and a neuronal network with a segmented distribution of cell bodies and axonal projections that traverse the CNS. While histological sections can provide 2D snapshots of these features, 3D imaging enables these anatomical features to be studied in their full, spatial context.

A variety of 3D soft tissue imaging methodologies exist. These differ in whether they are applied *in vivo* or *ex vivo*, in the spatial resolution they can attain, in the speed at which they can image large regions, in the tissue features that they emphasise, and how deep within samples they can probe. Synchrotron radiation X-ray microtomography (SRμCT) is a nominally non-destructive, rapid (sub-second to approximately one hour for data acquisition), high spatial resolution (effective pixel size ~0.2 to ~5.0 μm depending on magnification and detector) technique, able to image large (mm^3^) tissue regions with a large imaging depth and minimal tissue preparation. However, as specific labelling is not currently possible for SRμCT, it lacks the ability of traditional 2D histology to discriminate finer tissue features (e.g. cell subtypes and subcellular markers). Since SRμCT is a non-destructive 3D imaging methodology, this limitation could be overcome by its use in combination with subsequent 2D histology (or other methods) to derive maximal information from precious tissue samples (e.g. transgenic mouse strains which are difficult to breed, animal models of disease and pathology and post-mortem tissues). While examples of histology following soft tissue SRμCT exist^1–4^, no standard methodology has been developed to optimise this process.

**Figure 1:**
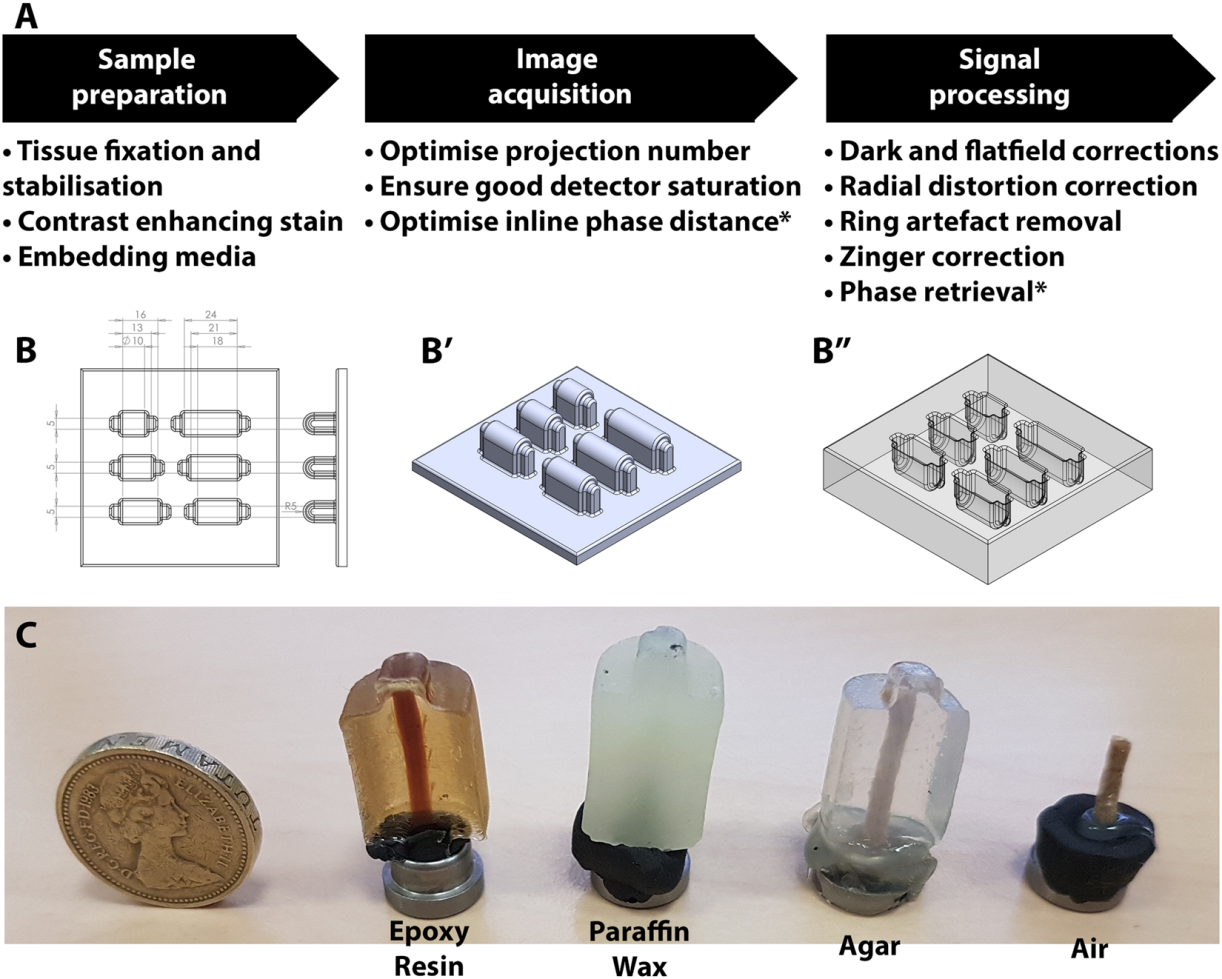
Synchrotron tomography optimisation and sample mounting. (**A**) Synchrotron radiation microtomography is a multi-stage technique containing multiple processes that affect the final quality and accuracy of image output. These can be categorised as sample preparation, image acquisition and sample processing. (**B**) Schematic (dimensions in mm) for the inverse block mould used for embedding linear tissue samples. (B’) Representation of the inverse block mould and the (B”) silicon mould cast from this. Resting the sample on the ‘lip’ of the mould ensures the central portion of the sample is surrounded by at least 3 mm of embedding material on all sides. Renders were made in Solidworks 2017 (Dassault Systèmes, France). (**C**) Examples of rat spinal cord embedded in four embedding materials; epoxy resin, paraffin wax, agar and air. Samples were fixed with plasticine onto metal ‘cryo cap’ bases which were attached via a magnet to a rotation stage. The air-mounted sample was additionally superglued to its base to aid stability.

SRμCT is a versatile technique, where the features revealed within tissue are determined by the tissue preparation and imaging strategy used. However, whether features are revealed at all is also a function of overall image quality. To ensure feature contrast is enhanced over noise, multiple aspects of SRμCT can be optimised. This can be thought of as a three-stage process, where cumulative improvements in sample preparation, image acquisition and signal processing enhance data accuracy and value (Fig. 1A).

Prior to SRμCT imaging, samples must be mounted for scanning. During scanning, samples are rotated across a range of angles to collect 2D projection images which are back-projected to form 3D tomograms. For high-resolution imaging, samples must remain physically stable during scanning – protected from both deformations and bulk movements that would compromise final tomogram quality. Soft tissues are prone to such instabilities, so benefit from stabilisation with fixatives such as paraformaldehyde or glutaraldehyde. Fixed soft tissues can be mounted and scanned in air^5,6^ or solution^7,8^, but more commonly are embedded in supportive matrices such as agarose gel^9,10^, paraffin wax^1–4,11–13^ or acrylic/epoxy resins^14–16^. These agents confer different levels of stability, but the choice of an extremely-stable resin is not a default, as it will limit how available tissues will be for analysis after SRμCT.

Amongst image acquisition parameters, the number of projections collected during tomography is a key determinant of data quality^17,18^. Too few projections will result in a low signal-to-noise ratio (SNR) with poor contrast, but too many incurs a time penalty that may expose samples to unnecessary and damaging levels of X-radiation^19^. Within SRμCT studies, the number of collected projections reported can vary widely. Various nervous system studies have collected 720^20,21^, 501–2000^22^, 1600^2^, 1800–2400^23^ and 2000–2400^3^ projections. The minimal number of projections that should be collected to avoid undersampling at the edge of the field of view is calculated by multiplying the pixel width of an imaging system (perpendicular to the rotation axis) by π/2^19^. However, increasing projections can improve on this minimal requirement^17^ and we outline a single-scan iterative downsampling methodology (adapted from a similar approach in cone-beam tomography^17^) to properly quantify how increasing projection number influences image quality and feature resolution.

Unmodified soft tissues are poorly X-ray absorbing, so will produce low absorption contrast. Contrast can be enhanced with contrast agents such as perfused radio-opaque plastics to emphasise vasculature^21,24,25^, or more generally by staining with contrast agents that bind to molecules (e.g. lipid or protein) within tissue (reviewed in Mizutani and Suzuki^26^). Lugol’s iodine (LI), phosphotungstic acid (PTA), osmium tetroxide (OsO_4_) and gallocyanine chromealum (GC), are all exemplar stains previously shown to improve X-ray contrast in various soft tissues^7,11,27,28^, but their application to CNS samples has not been directly explored. A confound to their use is that their introduction is usually irreversible, complicating subsequent use of tissue. The efficacy of these agents to enhance spinal cord contrast is compared here, along with a novel methodology to reversibly stain tissues with LI – preserving them for subsequent histology.

Soft tissue contrast can also be enhanced by utilising phase contrast. As X-rays pass through a sample, phase changes occur as X-rays pass across material boundaries (e.g. crossing a capillary). In a partially-coherent beam such as that of a synchrotron, as adjacent X-rays cross boundaries not perpendicular to the beam, they become out of phase and change direction leading to the formation of constructive-destructive interference Fresnel fringes. Practically, this leads to enhanced contrast of edge features and, following subsequent phase retrieval, an overall improvement in tissue contrast. Inline phase contrast is a simple phase imaging method in which contrast is increased by increasing the propagation distance (PD) between sample and detector (or scintillator, for a scintillator-coupled camera). It is unclear whether phase contrast can augment tissue stain contrast, but inline phase contrast of *unstained* samples has enabled visualisation of murine spinal vasculature^20^, large-diameter motoneurons^22,29^, cerebral vasculature^5^ and individual Purkinje cells in human post-mortem cortical tissue^3^. Details of how phase contrast was optimised have not commonly been reported, with PDs for nervous system samples varying from 300 mm^20^ to 500 mm^5^ and even 2,300 mm^22^. Determining the optimal PD is non-trivial, because if it is too small, contrast improvements will be insufficient to detect features of interest, but if it is too large, Fresnel fringes will grow to obscure a sample’s true profile, leading to inaccurate data. Optimal PD is therefore linked to the size of the features an investigator wishes to observe. It must balance enhanced visualisation of features of interest against the resolution loss and segmentation difficulties that can be associated with Fresnel fringes that arise during inline phase contrast imaging. Also, unlike absorption contrast, phase contrast voxel intensity is not linearly related to the density of the corresponding sample regions. Phase retrieval is necessary to restore, or partially restore, this property^30^.

A final aspect of enhancing image quality and data accuracy, is signal processing to correct for image acquisition artefacts. This includes zinger artefact reduction, dark- and flat-field correction, lens radial distortion correction^31^ and ring artefact suppression^32^. We demonstrate the use of such filtering following an evaluation of multiple sample mounting methods and sample contrast enhancing techniques. Many of these parameters have been evaluated previously in isolation, but our side-by-side comparisons help assess the relative and cumulative merits of each step to inform future SRμCT studies of the CNS and soft tissue tomography more generally.

Optimising multiple stages of SRμCT led to a wax embedded, LI stained, inline phase contrast setup that enabled efficient (~12 minutes/scan) imaging of the full width and depth of the spinal cord. Image quality improvements meant that the sample, white & grey matter and vasculature could be completely segmented across the 3D volume with the shallow machine learning Super Region Volume Segmentation (SuRVoS) Workbench while preserving tissues for subsequent histology.

## Results

### Comparing beam resistance of air, agar, wax and epoxy mounted samples

**Figure 2:**
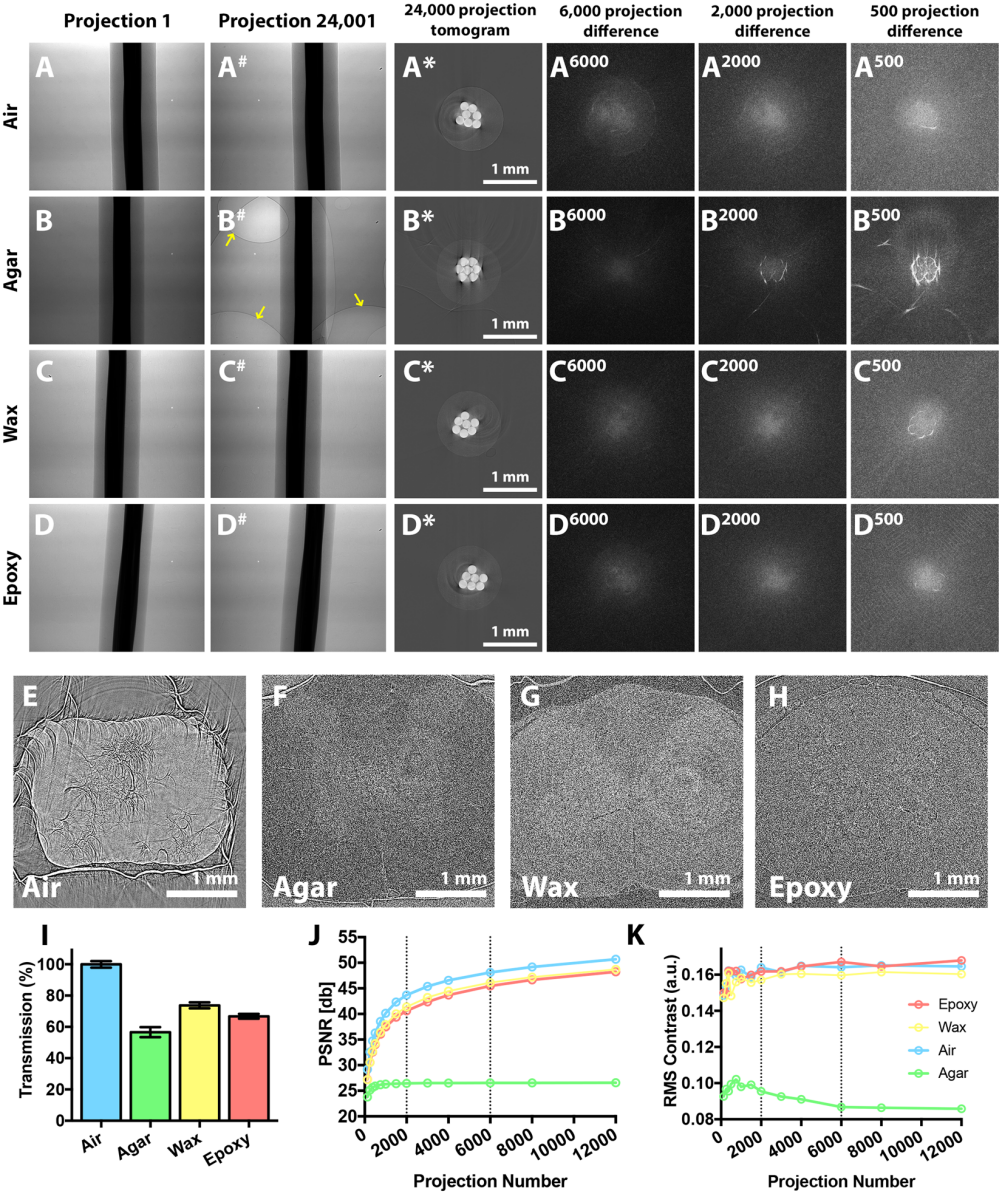
Testing embedding materials and optimising projection number. **(A–D**) Standard comparison samples made from embedding a piece of plastic-insulated wire containing seven drawn copper pieces in air, agar, wax and epoxy. These were subjected to an ‘over-projected’ ~40-minute scan (24,001 projections, 80 ms exposure). Comparing the **(A**–**D)** first and (A^#^,B^#^,C^#^,D^#^) last (24,001^st^) projection of each scan showed little deviation in air, wax and epoxy samples, confirming their stability during X-ray exposure. However, during agar scanning (**B**), expanding air bubbles (yellow arrows) shunted the wire piece. (A*,B*,C*,D*) Tomographic reconstructions were compromised in the (B*) agar-embedded sample by the sample being shifted by these expanding air bubbles. Difference maps between full-projection tomograms and tomograms reconstructed from 6,000 (A^6000^,B^6000^,C^6000^,D^6000^) 2,000 (A^2000^,B^2000^,C^2000^,D^2000^) and 500 (A^500^,B^500^,C^500^,D^500^) projection subsets. **(E**–**H)** 6,001-projection scans of a piece of unstained spinal cord embedded in each medium (propagation distance 20 mm) show that ‘air embedding’ does not adequately stabilise spinal cord samples. **(I)** X-ray transmission, assessed by comparing ten 100 × 100 pixel ‘background’ (embedding material only) regions in images of each embedding material to a flat-field image. Wax and epoxy absorbed ~30% of transmitted X-rays, with ~10% more absorbed by agar (mean ± standard error of the mean). **(J)** The peak signal to noise ratio (PSNR) measured in decibels [db] confirms that increasing projections reduces background noise, with ~85% saturation reached with 6,000 projections for wax. **(K)** The root mean square (RMS) contrast shows that increasing projections improves contrast, but this quickly saturates beyond ~2,000 projections.

To compare the suitability of various soft tissue SRμCT embedding media (air, agar, wax and epoxy) (Fig. 1C), standard comparison samples were made by embedding a piece of plastic-insulated copper wire in each material with our custom moulds (so ensuring samples had the same thickness). Stability in the beam was tested by exposing samples to an extended dose of X-rays (~40 minutes), while collecting 24,001 projections (80 ms exposure time). Comparing first and last (24,001^st^) projections of these scans revealed no detectable deviation in air (Fig. 2A,A^#^), epoxy (Fig. 2D,D^#^) or wax (Fig. 2C,C^#^) mounted samples, supporting their stability as embedding media. However, during agar sample scanning, expanding air bubbles shunted the wire, compromising tomogram quality (Fig. 2B,B^#^).

A comparison of unstained PFA-fixed rat spinal cord in the four media was performed to determine how media interacted with biological tissue. 6,001 projections (80 ms exposure time) were collected in ~12 minutes, emulating an X-ray dose expected in a real scanning scenario. No detectable deformations were noted in the epoxy (Fig. 2H), agar (Fig. 2F) or wax (Fig. 2G) embedded samples, but the air-mounted spinal cord had poor stability, drifting and deforming during scanning, so compromising final tomogram quality (Fig. 2E). Hence, air and agar were deemed sub-optimal embedding media for SRμCT scans.

Besides stability, comparing grey levels through media background within embedded-wire images to equivalent areas in flat-field images revealed a similar level of X-ray transmission through each embedding material (Fig. 2I). While epoxy and wax were both suitable for SRμCT mounting, wax was chosen for the remainder of this study because of its histology compatibility.

### Single-scan iterative downsampling to determine the optimal projection number to balance signal-to-noise with acquisition times

To establish the relationship between projection number and image quality, tomograms of the wire-embedded samples were reconstructed from the full 24,000 projection set and iteratively downsampled projection subsets. Increasing the projection number led to improved SNR as measured by PSNR (Fig. 2J) and contrast improvements as measured by RMS contrast (Fig. 2K). Contrast improvements peaked around 2,000 projections, but PSNR continued to improve with increasing projections. There was a sharp drop in image quality below 2,000 projections, so this was set as our minimal dataset for screening multiple imaging parameters (e.g. propagation distance). For exemplar scans of different contrast agents though, we collected 6,001 projections as we determined this gave a minimal time penalty for increased image quality.

### *Ex vivo* stains differentially improve tissue contrast

**Figure 3:**
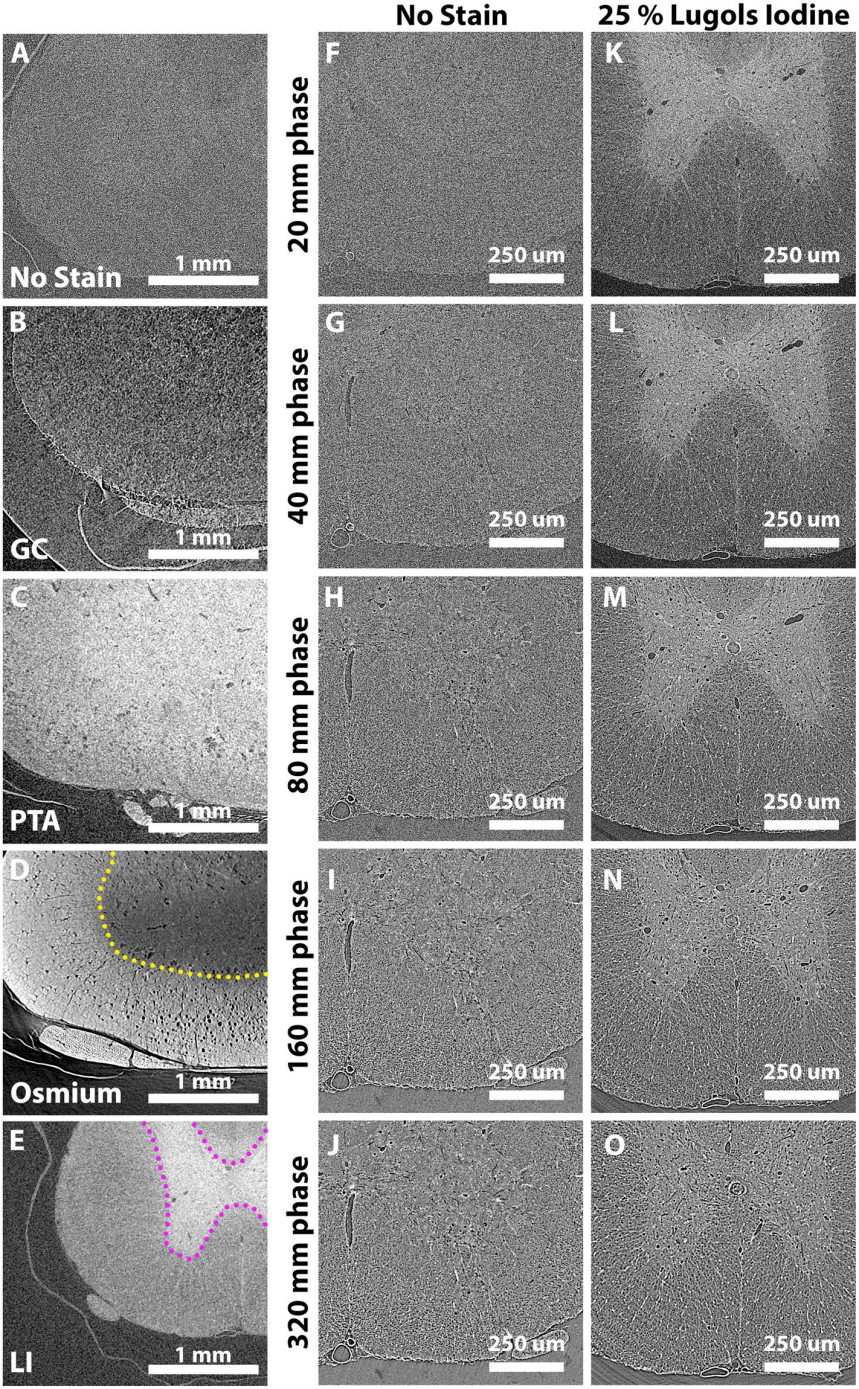
Enhancing sample contrast with sample stains and inline phase contrast. (**A**–**E)** Rat spinal cord samples stained with different heavy atom X-ray contrast agents and reconstructed from 6,000 projections with minimal phase contrast (20 mm PD is the minimum practical distance between the sample centre and the lens mounted scintillator to avoid collision during rotation) including **(A)** no stain **(B)** gallocyanine chromalum (GC) **(C)** phosphotungstic acid (PTA) **(D)** osmium tetroxide (OsO_4_) and **(E)** 25% Lugol’s iodine (LI). LI staining enhances grey matter contrast (pink border), while osmium staining enhances white matter contrast. However, osmium cannot penetrate to the centre of tissue, forming a barrier ~1 mm from the tissue edge (yellow border). **(F–O)** An unstained and LI-stained sample imaged with 2,000 projections at minimal PD (20 mm), 40 mm, 80 mm, 160 mm and 320 mm propagation distances (PD). Increasing PD increases tissue contrast in both samples, revealing tissue features including fine-diameter vasculature. LI-stained samples additionally exhibited white/grey matter contrast so were deemed relatively superior.

With X-ray absorption contrast alone, it was difficult to see unstained rat spinal cord (Fig. 3A). However, tissue contrast could be improved with *ex vivo* contrast agents^26^, four of which were evaluated. Despite earlier reports as a contrast agent that stains cell nuclei^7^, GC generated only marginal improvements in spinal cord contrast (Fig. 3B). PTA, which can bind protein and collagen^33^, contributed to improved tissue contrast across the sample (Fig. 3C). Osmium (which binds lipids) demonstrated the greatest improvement in tissue contrast (Fig. 3D), but this was restricted to the white matter perimeter by peripherally-deposited osmium forming a barrier that prevents osmium penetration to deeper tissue^23,34^. LI showed the greatest potential of the four stains tested. It led to an improvement in spinal cord contrast across the whole sample, particularly enhancing grey matter (Fig. 3E). Bright field imaging of serial sections from LI-stained whole spinal cords mounted in aqueous embedding media (so tissue can be visualised) emphasise contrast changes across whole tissue relative to unstained spinal cords (Supplementary Figure S1A,B).

Following LI staining, spinal cord length/width shrinkage of ~10–15% and a slight curling of some spinal cord segments was noted (Supplementary Figure S1H). However, this was also observed during dehydration of unstained tissue for paraffin embedding, suggesting iso-osmotic 25% LI does not impart extra disruption of tissue over paraffin embedding alone (Supplementary Figure S1C,D). Embedding also led to an ~50% increase in tissue density in both LI-stained and unstained tissue (Supplementary Figure S1E–G), which may explain increased contrast in paraffin wax embedded tissue (Fig. 1G) over hydrated agar-embedded samples (Fig. 1F), as previously suggested^6,35^.

### Inline phase contrast imaging enhances spinal cord contrast and augments tissue staining contrast

Besides contrast agents, inline phase contrast can enhance contrast of samples with poor X-ray absorbing properties; the extent of which is determined by the PD. The optimal PD for unstained and LI-stained rat spinal cord samples was determined by screening a single sample of each preparation at multiple distances between 20 and 320 mm (Fig. 3F–O). Increasing PD revealed internal features such as the vasculature within unstained tissue (Fig. 3F–J). The same features were revealed with greater clarity alongside the white-grey matter boundary in the LI-stained tissue (Fig. 3K–O). 160 mm seemed the optimal PD, revealing microstructural features including capillary-level detail and motoneurons (Fig. 3N). At 320 mm and beyond, these features were obscured by overly edge-enhanced features (Fig. 3O). Hence, 160 mm was deemed the optimal PD for our embedded spinal cord samples.

**Figure 4:**
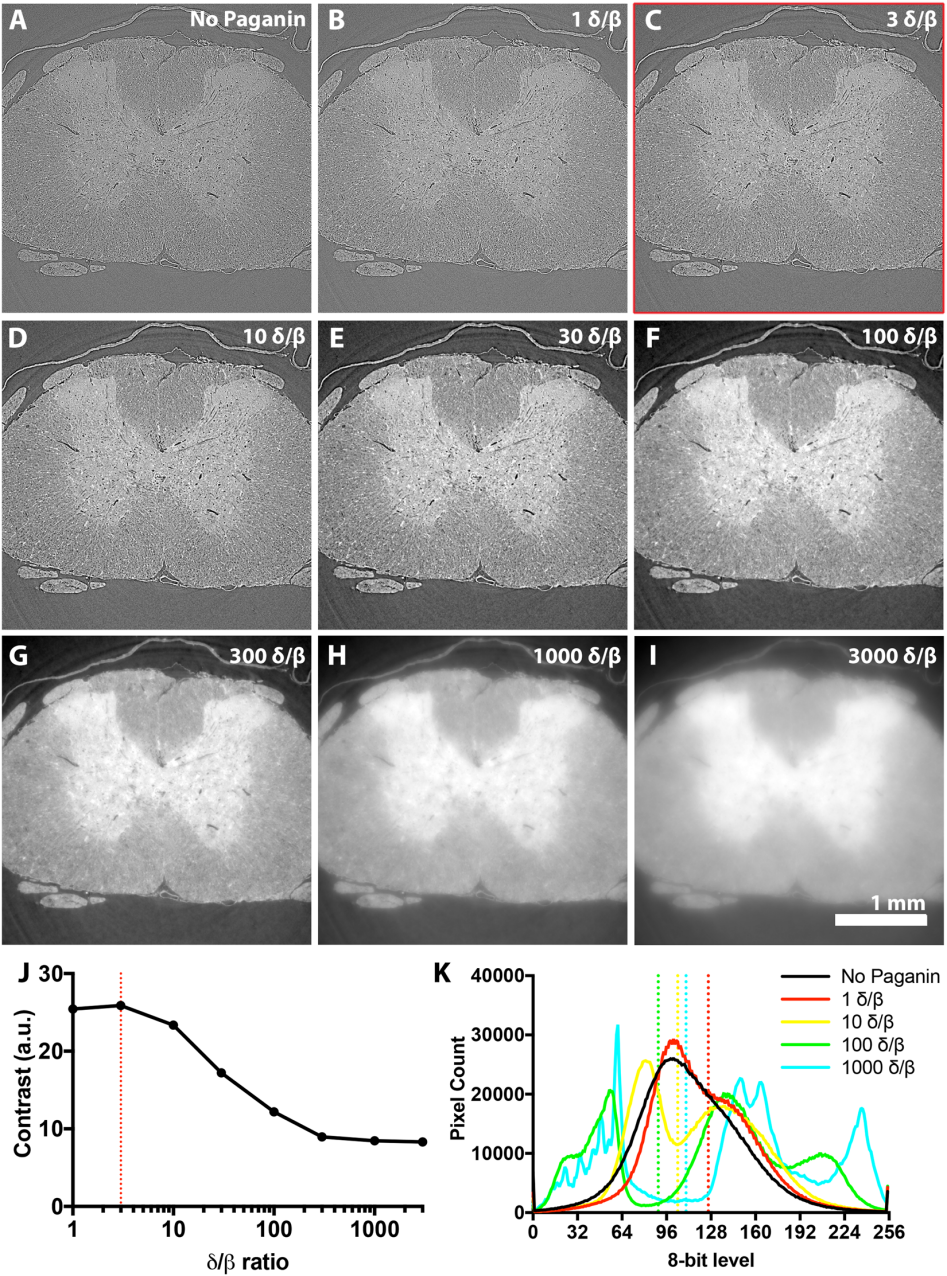
Optimising phase retrieval of phase contrast images with Paganin filtering. The degree of phase retrieval needed depends on the relative contribution of phase shift (𝛿) over absorption (β). As this is related to feature of interest size, chemical composition and homogeneity of the sample, for a heterogeneous biological tissue like the spinal cord 𝛿/β is best determined empirically. **(A–I)** 25% LI-stained spinal cord imaged with 160 mm PD with either **(A)** no filtering, (**B)** 𝛿/β = 1, **(C)** 𝛿/β = 3 **(D)** 𝛿/β = 10, **(E)** 𝛿/β = 30, **(F)** 𝛿/β = 100, **(G)** 𝛿/β = 300, **(H)** 𝛿/β = 1000 or **(I)** 𝛿/β = 3000. **(J)** Plotting RMS contrast demonstrates slight contrast enhancement at 𝛿/β = 3, but contrast loss beyond 𝛿/β = 10. **(K)** Comparing whole tomogram slice grey level histograms of unfiltered and Paganin-filtered images reveals peaks introduced following filtering; these aided segmentation of sample (higher peak) from background (lower peak). Dotted lines denote background cut-off which is still ambiguous if too low a level of Paganin filtering is applied.

Phase retrieval of inline phase contrast scans was performed with Paganin filtering to restore quantitative detail to tomograms^36^. The degree of Paganin filtering is proportional to the contribution of X-ray absorption (β) and phase shift (𝛿) to projections. Under-filtering conferred marginal benefit (Fig. 4B), but over-filtering led to feature loss and blurring (Fig. 4E–I). A low to moderate degree of filtering gave the best results (Fig. 4C,D). A RMS contrast comparison confirmed slight contrast improvements at 𝛿/β = 3, with loss beyond 𝛿/β ~10 (Fig. 4J). Paganin filtering also improved grey level separation of sample and background (Fig. 4K).

### Zinger and optical distortion correction improve data accuracy

Data accuracy can be improved by correcting for artefacts introduced during acquisition. These include ‘standard’ approaches such as dark and flat-field corrections and ring artefact reduction, as well as zinger removal and radial distortion correction. Zingers result from stray X-rays and cosmic rays striking the detector’s sensor during image acquisition^37^. In projection images these manifest as narrow (a few pixels), linear streak artefacts, while in and dark- and flat-field images, they present as ring artefacts. Though subtle, zooming to pixel level reveals ~20 zingers per tomographic slice (Supplementary Figure S2A”,A*,B”,B*). These can be filtered out, with difference maps between uncorrected and zinger corrected tomographic slices emphasising their presence (Supplementary Figure S2C,C’,C”).

**Figure 5:**
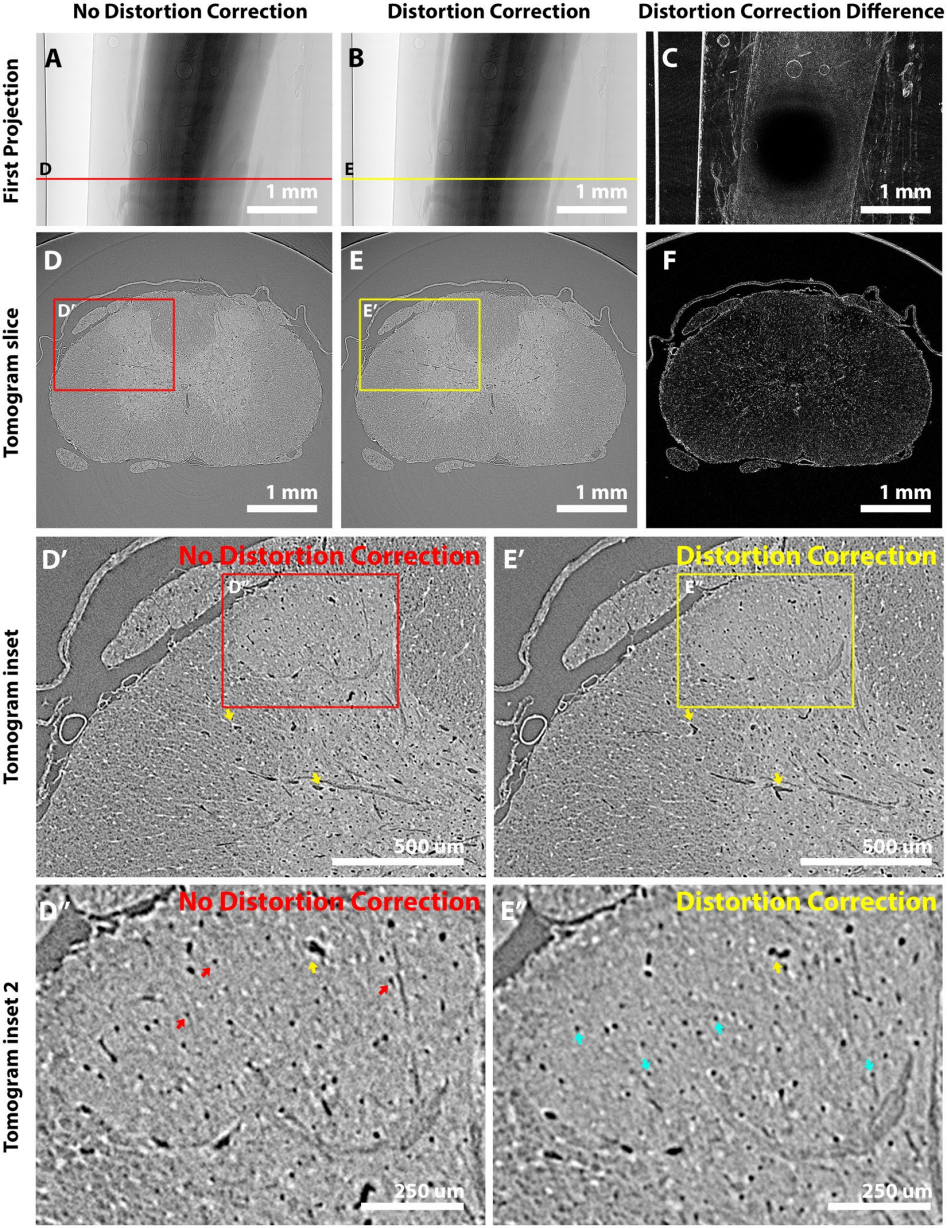
Radial distortion correction to improve data accuracy. Microscope lenses introduce radial distortion inaccuracies to data which compromise accuracy. Distortion corrections can be calculated from a standard grid pattern and then applied to collected images. The first projection (**A**) without and (**B**) with distortion correction; (**C**) the difference between (**A**) and (**B**) demonstrates how distortions are greatest towards the edges of the FoV. A tomographic slice constructed from zinger, flat-field and dark-field corrected images (**D**) without and (**E**) with distortion correction; **(F)** the difference between (**D**) and (**E**). (D’,E’) Insets demonstrate how fine features such as vasculature (yellow arrow examples) can be lost or shifted in/out of plane without distortion correction. (D”,E”) At higher magnification, in the dorsal horn of the grey matter, capillary-level vasculature can be seen moving in/out of the plane. Radial distortion correction also helps to reduce centre-of-rotation errors towards the edge of the FoV that distort otherwise uniform circular capillaries (red arrows). The finest features that can be identified are capillaries of 4–5 pixel widths (blue arrows showing examples) corresponding to vasculature ~4.8 to 6.4 μm in diameter.

Radial distortions introduced by lenses used during imaging contribute yet greater data artefacts. Calculated difference maps between a non-distortion-corrected projection (Fig. 5A) and a distortion-corrected projection (Fig. 5B) emphasise this (Fig. 5C) and highlight how the greatest differences are present towards the edge of the field of view (FoV). This translates to differences in tomograms reconstructed from non-distortion-corrected projections (Fig. 5D) and distortion-corrected projections (Fig. 5E,F), with pixel-level shifts introducing data inaccuracies that bring different aspects of fine vasculature into and out of a tomographic slice (Fig. 5D’,E’). Distortion corrections also help to reduce ‘crow’s feet’ artefacts (Fig. 5D”,E”) found towards the edge of the FoV, so features such as capillaries regain their signature rounded morphology.

**Figure 6:**
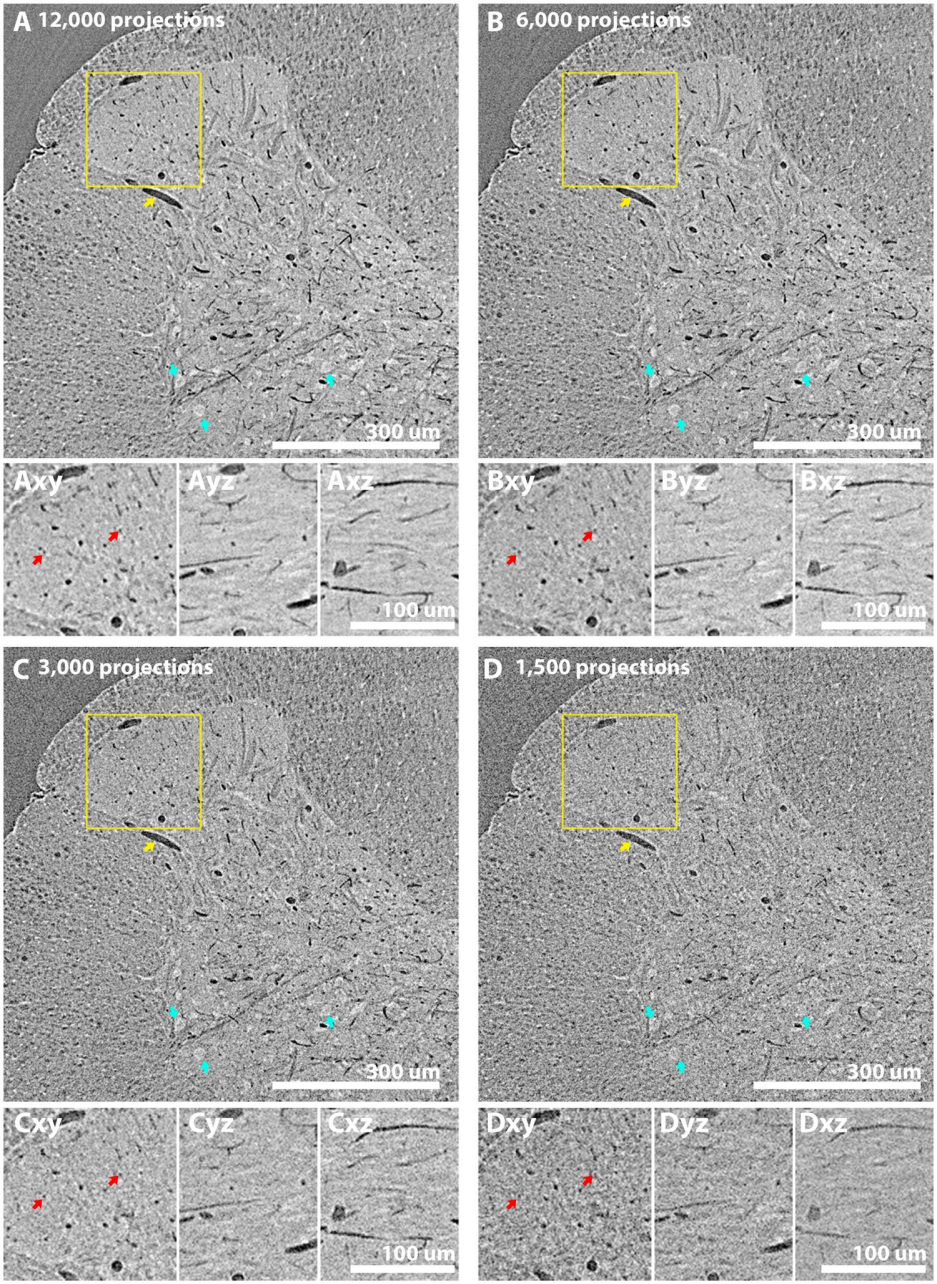
Large features are resolved with low projections numbers, but increasing projection numbers aids resolution of micron-level features such as capillaries. A 12,001-projection scan collected with our optimised protocol (25% LI stain, paraffin wax embedded, 160 mm propagation distance, artefact corrected) was iteratively downsampled by a factor of 2 to assess the contribution of increasing projection number to tissue feature observation. All images are minimum intensity projections of 2 slices (effectively becoming ‘3.2’ μm thick slices) to emphasise the darker vasculature. In the (**A**) 12,000 (**B**) 6,000, (**C**) 3,000 and (**D**) 1,500 projection datasets, large features such as major vasulature branches (yellow arrow indicates a large ~13.2 μm diameter piece of vasculature) and large diameter motoneurons (blue arrows indicate motoneurons ~24 μm in diameter) could be resolved. With higher projection-number tomograms (12,000 and 6,000), resolution of finer features including capillary-level vasculature ~2–3 pixel widths (~3.2 to 4.8 μm wide) in diameter could be seen, as confirmed with re-sliced tomogram insets.

Following contrast enhancement and artefact correction, fine capillaries ~4 pixels wide (6.4 µm diameter) could be observed within our parameter screening, 2,000-projection tomograms (Fig. 5E”, blue arrows). To assess whether increasing projections improve feature resolution, a 12,000-projection scan of an LI-stained spinal cord was collected with our optimised protocol (25% LI stain, paraffin wax embedded, 160 mm propagation distance, artefact corrected) and reconstructed (Fig. 6A) alongside iteratively-downsampled 6,000 (Fig. 6B), 3,000 (Fig. 6C) and 1,500 (Fig. 6D) projection tomograms. While large motoneuron cell bodies (cyan arrows in Fig. 6) 20–30 μm in diameter, could be observed across all datasets, fine capillary structures (confirmed with resliced tomograms in Fig. 6 insets) only 2–3 pixels in diameter (~3.2–4.8 μm wide) were only obvious with 12,000 and 6,000 projection datasets.

### Volume segmentation of sample, white and grey matter and vasculature with SuRVoS

**Figure 7:**
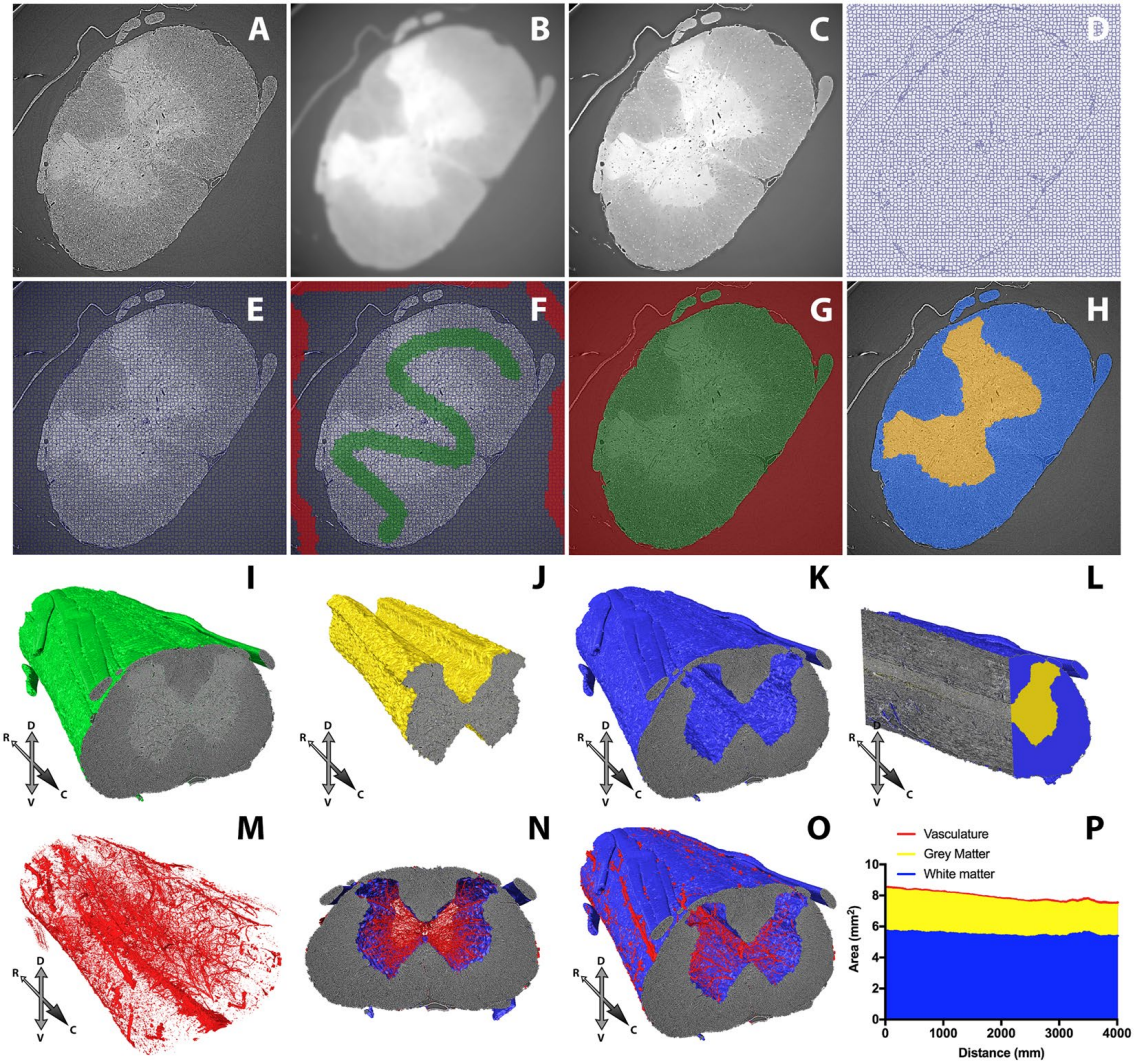
Segmentation of the low thoracic-high lumbar (T13-L1) level spinal cord sample from background, white & grey matter from spinal cord and vasculature from spinal cord with SuRVoS. (**A**) Distortion-corrected tomograms from wax embedded, LI stained, phase contrast imaged spinal cord. Tomograms processed with (**B**) Gaussian and (**C**) anisotropic total variance filters emphasise macro features within tomograms. (**D**,**E**) Supervoxels mapped to filtered data with compactness adjusted to follow edges. (**F**) Manually annotated supervoxels to demark sample and background are used to predict sample and background regions. Model training is refined to (**G**) segment spinal cord sample from background. The same process can hierarchically segment (**H**) white and grey matter within this spinal cord sample region. (**I**) Segmentation of whole spinal cord volume, (**J**) grey matter volume and (**K)** white matter volume. **(L**) White and grey matter volume bisected longitudinally. (**M**) Spinal cord vasculature segmented by grey level thresholding within the spinal cord volume from (**I)**. (**N**) Spinal vasculature overlaid on grey matter volume in transverse orientation emphasises radial centripetal vasculature. (**O**) Vasculature overlaid on white matter also demonstrates surface vasculature. (**P**) Area measurements of white matter, grey matter and vasculature within total spinal cord volume. Compass abbreviations; (R)rostral, (C)caudal, (D)dorsal, (V)ventral.

With SRμCT scanning parameters optimised and artefacts corrected, various macro- and micro-features could be discerned within spinal cord samples. Segmentation of these features was achieved with shallow machine learning through the SuRVoS Workbench^38,39^. Features within unbinned tomograms (Fig. 7A) were emphasised through 3D Gaussian (Fig. 7B; 3-pixel radius) and anisotropic total variance (Fig. 7C; 3-pixel radius) filtering. 3D supervoxels were then mapped to these filtered datasets with deformability (‘compactness’) adjusted so that supervoxels followed the contours of features of interest (Fig. 7D,E). Coarse manual annotation of supervoxels within two classes (sample and background) for ~10 slices evenly spaced throughout the full 3D volume (Fig. 7F) were used as training data for class prediction (extra random forest algorithm with edge refinement) for all other supervoxels in the volume. These predictions were refined through further manual annotation and correction of erroneous predictions within SuRVoS. This enabled sample segmentation from background across the entire imaged volume (Fig. 7G,I and Supplementary Video 1). White and grey matter were then hierarchically segmented within the spinal cord volume using the same strategy (Fig. 7H,J–L, Supplementary Video 2). Finally, vasculature was segmented via grey level thresholding in the grey and then the white matter volumes, before combining to total vasculature (Fig. 7M–O). The large anterior spinal artery, along with central arteries that sprout from this to the central grey matter were apparent, alongside the finer capillary network within the cord parenchyma. Measurement of the complete white matter, grey matter and vasculature areas along the rostro-caudal axis could then be achieved (Fig. 7P).

### Reversible iodine staining for subsequent histology of SRμCT-imaged tissues

**Figure 8:**
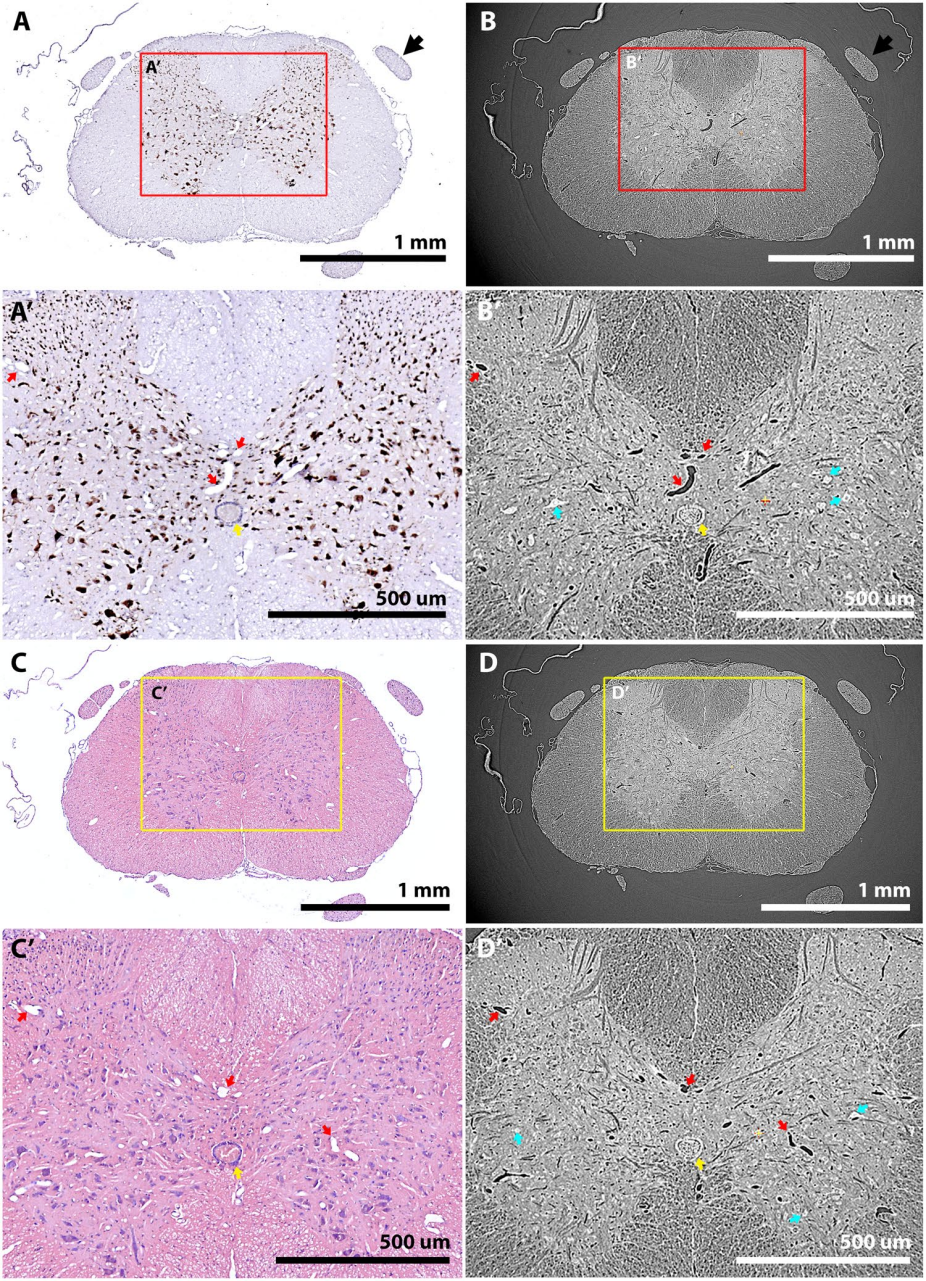
Wax-embedded tissue imaged by SRμCT is suitable for subsequent histology and the resulting images can be aligned to tomograms. (**A**,A’) NeuN immunostaining of 7 μm thick transverse tissue sections demonstrate that immunolabelling of tomographically-imaged tissue is also possible. (**B**,B’) A ‘6.4 μm’ tomographic slice (mean z-projection of 4 optical slices to match mechanically sectioned, 7 μm slice) matched to the NeuN–stained image in (A) with consistent morphological features including vasculature (red arrows) and the central canal (yellow arrow). At low magnification, dorsal roots are also seen to align (black arrowheads in A, B). At higher magnification, some motoneuron cell bodies (cyan arrows) can be identified in tomograms. (**C**,C’) Haematoxylin and eosin staining of 7 μm thick transverse tissue sections reveals that histological staining of tomography tissue is also possible. (**D**,D’) A ‘6.4 μm’ slice from a matched tomogram, again showing consistent features including vasculature (red arrows), the central canal (yellow arrows) and large motoneurons (cyan arrows).

Following CT imaging, tissue could be sectioned for subsequent histology. During sectioning, it was found iodine perturbed tissue sections adhering to charged slides, likely due to iodine affecting charge within tissue. A method for removing iodine from samples by incubating dewaxed samples in sodium thiosulphate (used to remove iodine in gram-staining^33^) overcame this limitation. Tissue suitability for immunohistology was demonstrated through NeuN immunolabelling to identify all neuronal cell bodies throughout the spinal grey matter (Fig. 8A,A’), and with standard histological stains such as hematoxylin and eosin (Fig. 8C,C’). Both histological preparations could be aligned to their respective tomographic slices (Fig. 8B,B’,D,D’). However, these did not completely overlap, likely due to a combination of sections being off-plane, tissue being shifted during re-embedding and some mechanical distortion of tissue during sectioning. Nevertheless, tissue was viable for analysis by traditional 2D histology and fine common features could be readily identified in image pairs (Fig. 8C’,D’).

## Discussion

Optimising tissue preparation and imaging parameters transforms SRμCT into a high-speed, high-resolution, genuinely non-destructive soft-tissue imaging technique. When applied to CNS samples, SRμCT can resolve macro and micro features including white-grey matter, the central canal, vasculature and large motoneuron cell bodies. Some of these features have been revealed by SRμCT before, but we demonstrate how optimising sample preparation and SRμCT parameters can reduce scan times and achieve enhanced resolution and contrast (aiding features visualisation and segmentation) across extended tissue regions, while preserving tissues for subsequent histology.

We evaluated several options for preparing and mounting CNS samples and determined that wax and epoxy had optimal mounting properties for SRμCT imaging. They also offered practical benefit, as tissues embedded in either material are physically protected during handling and can be stored at room temperature for long periods of time. This is advantageous as samples can be prepared well ahead of valuable beam time to accommodate potential delays. Both wax and epoxy are also low cost and simple to use, with many research groups having protocols in place for their routine use in histology and electron microscopy (EM) preparations, respectively.

For wax-embedded samples, beam damage was reduced to acceptable levels by undulator gap adjustment, high-pass filtration and minimising projection number to maintain a reasonably good SNR without over-exposing samples. If more physically-stable samples are required such as for repeat imaging or long duration imaging at high magnification (thinner, less efficient scintillators must be used to achieve high resolution with high magnification objectives), epoxy resin offered ultimate sample stability. The resin formula we utilised was adapted from EM protocols^40^ so EM could theoretically follow SRμCT, as previously demonstrated^23^. However, resins preclude the use of tissue for most subsequent histology techniques and if these were required, wax embedding would be the method of choice. Wax has some compromises, with small angle X-ray scattering by wax crystals^41^ contributing to slight signal blurring during imaging. However, this did not undermine feature observation within our samples, or in earlier demonstrations successfully combining paraffin-based histology with SRμCT to image human cerebellar^3,4^ and soft vascular tissue^1^. The complementarity of paraffin may in future allow SRμCT to be combined with techniques such as mass spectroscopy^42,43^ or RNA sequencing^44^, both of which have already been successfully used on wax embedded tissues.

Projection number influences image quality and the efficiency of image acquisition. As stated, the minimal number of projections that should be collected to ensure proper sampling at the edge of the full FoV can be calculated – in our instance being ~4021 (2560 * π/2) projections. Our observation that ~2,000 projections was sufficient for good image quality during parameter screening may be due to our samples being restricted to just over the central 50% (1300 * π/2 = 2042 projections) of the FoV. This highlights how the *minimal* number of projections for proper sampling is also related to sample size. In terms of the *maximal* number of projections that should be collected though, our iterative downsampling methodology helps to reveal the relationship between increasing projection number and improvements in image quality and feature resolution. These improvements must be balanced against associated penalties – principally acquisition time, but also slower reconstruction speeds (due to increased computational requirements) and increased data storage (due to increased number of projections). Our iterative downsampling methodology helps to minimise these as per the needs of an experiment. In our instance, it revealed that ~6,000 projections provided a good SNR and capillary-level resolution without incurring excessive penalties.

At 4x total magnification, 6,001 projections of a 3.5 mm length of spinal cord were acquired in ~12 minutes. At this rate, a complete rat spinal cord (~10 cm in length) could theoretically be completely imaged with ~30 concatenated scans (including a 2% overlap) at a resolution that allows fine capillaries to be resolved in ~6 hours. Comparable methods such as *ex vivo* diffusion tensor imaging achieve lower resolution (mm) imaging of thin (~1 mm thick) sections of rodent spinal cord (around one third the volume imaged here) with less contrast even when using extensive *12–13 hour* data acquisition protocols^45,46^. Such a speed advantage widens the scope of SRμCT applications, as it becomes feasible to image extended tissue regions, or image large sample sizes (such as a pathological time course in models of CNS injury or disease or different treatment groups) in a practical time frame.

It is not commonly reported how inline PD and phase retrieval levels were determined for inline phase contrast CT imaging. One prior study reported scanning mouse brain samples at 100 mm PD intervals between 100 and 1100 mm, determining by eye that ~700 mm was the optimal PD^2^. We judged the optimal PD of wax embedded spinal cords to be ~160 mm. Optimal PD varies according to the size of features to be observed and the photon energy of the X-ray beam^36^. If these are known values, a range of suitable PDs can be calculated theoretically (see Equation 1)^30^. 3.2 μm diameter capillaries and our peak beam energy (25 keV), suggests that a maximum PD for these features would be ~207 mm, with 160 mm representing the maximum suitable PD for features ~2.82 μm in size. At 160 mm, phase contrast of features larger than 2.82 μm would not be maximised, while features smaller than this may be obscured by Fresnel fringes. As imaging was performed at 4x total magnification, features smaller than 2.82 μm would be fewer than 2 pixels (2.43 μm) wide, so may struggle to be resolved anyway. With our set-up, 160 mm PD may therefore represent a point where features smaller than 2.82 μm are blurred (but would have been lost anyway), but larger features are enhanced (though not maximally) without being obscured. In future studies with samples containing unknown (or a range of) features sizes, a starting point for estimating optimal PD might be to screen around the theoretical optimal PD for 1–2 times the magnified effective pixel width of the detector.

1 $$PD\le {a}^{2}/\lambda $$

Fresnel regime relationship to estimate suitable propagation distances (PD) for different feature sizes. a is feature diameter and λ is X-ray wavelength.

Of the contrast-enhancing stains evaluated, LI and OsO_4_ provided the best tissue contrast. OsO_4_ is well recognised for its lipid-binding abilities, but beyond binding of some amino residues^33^, the exact targets of LI within tissue are unclear. Though we demonstrated iodine-enhanced contrast of tissue to help differentiate grey and white matter, future work on the specific chemistry of tissue binding by iodine will help with understand of its use as a contrast agent and may reveal better stain alternatives.

LI in combination with phase contrast enabled identification of capillary level vasculature and large neuronal cell bodies in the ventral and intermediate lamina. Motoneurons have been observed previously in the ventral horns of the mouse spinal cord by inline phase contrast SRμCT^22,29^, but our optimised preparation has enabled higher contrast and higher resolution so that they can be more clearly distinguished – particularly large-diameter motoneurons. Smaller neuronal cell bodies such as those in the superficial lamina of the dorsal horn were difficult to distinguish, though this may be possible at higher magnification (not assessed here as the associated FoV would not encompass the whole spinal cord). Interestingly, there is some variability in the levels of contrast exhibited by large motoneurons. It is unclear why this might be, but it could be due to the different physical properties of certain motoneuron subtypes (e.g. alpha vs. gamma motoneurons)^47^.

While LI improved tissue contrast, it also led to some tissue shrinkage. This does not change relative features within samples, but does mean that care should be taken when making morphological comparisons between samples. LI-staining parameters, including stain duration, concentration and washout have been optimised in other tissues to minimise these effects^7,11,27,48^. Indeed, disruption between unstained and iso-osmotic 25% LI stained tissue was comparable following embedding, with shrinkage levels similar to previous descriptions after paraffin embedding (5–15% depending on tissue type)^49^. Nevertheless, if *ex vivo* tissue stains needed to be avoided, imaging with phase contrast alone is sufficient to reveal many soft tissue internal structures (albeit with less contrast).

The enhanced contrast of the spinal cord specimen and internal macro-features within tomograms enabled volume segmentation of white matter, grey matter and vasculature across the entire scanned volume. Grey level inconsistencies and size variance of neuronal cell bodies complicated their semi-automated extraction with our shallow machine learning approach, though deep neural networking may be able to overcome this challenge. Nevertheless, the current extraction of macro-features has applications for tracking gross morphological changes which are present following multiple spinal cord pathologies including spinal cord injury, cord compression syndrome, cord inflammatory diseases, spinal ischaemia, amylotrophic lateral sclerosis and cervical spondylotic myelopathy^50^. These could then be more closely investigated with subsequent histology.

Optimising sample preparation, imaging parameters and signal processing improves the quality of 3D data that can be derived from biological samples by SRμCT. Our side-by-side comparisons of how these parameters contribute to tissue sample contrast and image quality are a useful reference for how to derive excellent CNS contrast and SNR, while methods outlined to optimise scanning parameters and mount samples for either ultimate stability (epoxy) or reversibility (wax) have application to soft tissue tomography generally (summarised in Table 1). Together, improvements in sample preparation, data collection and processing strategies make segmentation and analysis more efficient and effective.

**Table 1:** Summary table of those stages evaluated for *ex vivo* soft tissue imaging by SRμCT.

SRμCT Stage		Individual Parameter								
Sample preparation	Embedding media	***Epoxy*** *Excellent stability, Non-reversible*		**Wax** Good stability, reversible		**Air** Does not stabilise samples			**Agar** Poor beam resistance	
	Tissue stain	**Lugol’s iodine** Whole tissue white-gray matter contrast	***Osmium tetroxide*** *Excellent white matter contrast, but poor tissue penetrance*		***Phosphotungstic acid*** *General tissue contrast increase*			**Guanidine chromealum** No benefit		***Unstained*** *Avoids possible stain confounds and is suitable for phase contrast*
Imaging parameters	Projection number	**<2000** Low quality final tomograms		2,000–10,000* Good quality final tomograms					*>* ***8,000*** *Excellent quality final tomograms, but time penalty*	
	Inline phase contrast propagation distance	<**40** **mm** No phase contrast benefit	***80*** ***mm***			**160** mm*		***320*** ***mm***		>**320 mm** Fresnel fringes obscure fine features
Signal processing	Phase retrieval	**None** Phase retrieval necessary to minimise phase artefacts		<***1 δ/β*** *Too low, no measureable effect*			**1–10** ***δ*** /β* Fine feature preservation with grey level feature separation		>**10** ***δ*/β** Too much, blurs fine feature detail	
	Artefact correction	Zinger removal		Dark- and flat-field			Radial distortion		Ring suppression	

Underlined are those options which were optimised for complementary SRμCT. Those processes marked with an asterisk (*) may vary for other experiments depending on the sample size and imaging conditions (see text for detail), with the reported values being optimal in our case. Italicised options could also be utilised for SRμCT with certain considerations (e.g. epoxy embedding offers excellent stability but not reversibility, unstained tissues can be visualised under phase contrast conditions, or more projections can be collected to further improve SNR).

## Materials and Methods

### Animals and tissue collection

All animal work was carried out in accordance with UK Home Office legislation (Scientific Procedures Act 1986) and approved by the King’s College London Animal Care and Ethics Committee. Four adult male Lister Hooded rats (~350 g; Harlan, UK) were deeply anesthetized with sodium pentobarbital (Euthatal, 80 mg/kg, administered intraperitoneally) and transcardially perfused with phosphate buffered saline (PBS with 0.01% heparin) followed by 400 ml of 4% paraformaldehyde (PFA; in 0.1 M PBS, pH 7.4). Cervical (~C2) to lumbar (~L5) spinal cord tissue was dissected and post-fixed overnight in this solution, and then washed 3 × 1-hour in PBS. Post-fixed spinal cord was divided into ~8 mm sections which were then separated for staining/no staining before embedding.

### Tissue staining

5% GC was prepared from a 5% (m/v) chromealum and 0.15% (m/v) gallocyanine stock in H_2_O^51^. An iso-osmotic (308 mOsm/L), 25% LI solution (diluted in H_2_O) was prepared from a 100% LI stock (10 g KI and 5 g I_2_ in 100 mL H_2_O)^11^. Tissues were stained for 48-hours in 5% GC, 25% LI or 2% osmium tetroxide (OsO_4;_ Electron Microscopy Sciences #19170). Excess stain was removed with 3 × 24-hour PBS washes and tissues were then dehydrated through 8-hour industrial methylated spirits (IMS) incubations (30, 50 and 70%) before embedding. For PTA staining, tissue was *first* dehydrated through 8-hour IMS incubations (30, 50 and 70%), and then stained for 48-hours in 2.5% PTA (5% PTA hydrate v/v in 70% IMS). Tissue was gradually dehydrated prior to PTA staining as placing hydrated soft tissue in PTA can lead to sample cracking through rapid dehydration^7^. Excess PTA stain was removed with 3 × 24-hour 70% IMS washes before embedding.

### Tissue mounting and embedding

All tissues were embedded in custom cylindrical silicon moulds cast from a 3D-printed template (Fig. 1B) designed in Solidworks 2017 (Dassault Systèmes, France) and available at https://www.thingiverse.com/thing:2835786. During embedding, resting samples on the raised lip of these moulds ensured that a central portion of tissue was surrounded by ~3 mm of material on all sides. This was necessary to avoid streak artefacts arising from strong Fresnel fringes at interfaces between embedding materials and air. For agar, post-fixed tissues were embedded using 2% high melting point agarose (Sigma, #A7174) and then stored in PBS at 4 °C until imaged. For wax, dehydrated tissues (in 70% IMS) were embedded using a tissue processing station (Leica TP1020). Tissues were completely dehydrated (2-hour washes in 90% IMS and 3 × 100% IMS), cleared (2 hours in Xylene-IMS 1:1 and 3x Xylene), then wax infiltrated (2 hours in 2x Paraffin wax) and wax cast. For air and Spurr’s epoxy^52^ embedding, tissues were completely dehydrated with 8-hour incubations in IMS (30, 50, 70, 90 and 100%). ‘Air-embedded’ tissues were stored in 100% IMS until imaging. Epoxy-embedded tissues were moved through 24-hour IMS:propylene oxide incubations (30, 50, 70, 90 and 100%) and then infiltrated with a 24-hour propylene oxide: epoxy series (30, 50, 70, 90 and 100%) on a low-speed vortex. Tissues were then epoxy cast at 60 °C for 48 hours.

### Quantifying tissue parameter changes during 25% LI staining and embedding, relative to unstained embedding

After post-fixation, two adult male rat spinal cords were each divided into 4 equal length segments ~20 mm in length. These segments spanned either the upper cervical enlargement, upper thoracic, lower thoracic or lumbar region of the spinal cord. Every other segment was separated for either the 25% LI staining procedure, or to remain unstained. Sections from each spinal cord were mismatched to treatments so that each cord segment was represented by both procedures (Supplementary Figure S1H). The stained and unstained segments were then manually prepared for paraffin embedding (rather than using the tissue processing station) using the same incubations as outlined above. Following post-fixation and after each step of the staining and embedding procedure, segment length and width (at the widest lateral point) were measured with digital callipers accurate to 0.01 mm (#501601, World Precision Instruments, USA). Mass changes and volume displacement were also measured and used to calculate tissue density changes during embedding.

### Wax tissue iodine removal and histology

To enable subsequent histological assessments following SRμCT imaging, it was necessary to first remove iodine from the tissue. Wax-embedded LI stained tissues were dewaxed by heating to 60 °C and passing through 2 × 8-hour Xylene washes. Tissues were then rehydrated through 8-hour IMS:H_2_O incubations (100, 70, 50, 30 and 0%) and placed in 5% (m/v) sodium thiosulphate (in H_2_O) overnight to remove iodine. Tissues were then washed in PBS, dehydrated and re-embedded in wax (as above). Wax blocks were sectioned on a microtome into 7 μm-thick sections and mounted onto glass slides.

For haematoxylin and eosin staining, mounted sections were stained as detailed previously^53^. Following antigen retrieval, NeuN immunostaining was performed as detailed previously^54^. Briefly, dewaxed slides were placed in a pressure cooker with 1% citrate at pH 6.04, rinsed with tris buffered saline (TBS), then incubated in the following (with TBS rinses between each stage): 1% bovine serum albumin (in TBS pH 7.6, 1 hour), mouse biotinylated-NeuN antibody (Millipore MAB377; 1:500 in block solution, overnight), StreptABC-HRP (Vectorlabs PK6100; 1:400, 1 hour) and 3,3-diaminobenzidine tetrahydrochloride (Sigma D5673; 10% in TBS, 30 minutes). Slides were then TBS rinsed, counterstained with haematoxylin, dehydrated, xylene-cleared and cover-slipped in DPX. All slides were imaged under a bright field Zeiss AxioCam.

### Sample mounting and tomography

Tomography was performed at the Diamond-Manchester Imaging Branchline I13–2 of the Diamond Light Source (DLS) synchrotron (Oxfordshire, United Kingdom)^55,56^. A partially-coherent, near-parallel, polychromatic ‘pink’ beam was generated by an undulator in an electron storage ring of 3.0 GeV voltage and 130 mA current. The ring normally operates at 300 mA, with the lowered current in this instance arising from operation in ‘156 bunch’ mode. For data collection, the undulator gap was set to 5.8 mm. Although flux can be increased by reducing the gap to its minimum of 5.0 mm, this was found to result in paraffin damage, particularly with repeat scanning. The beam was reflected from the platinum stripe of a grazing-incidence focusing mirror and high-pass filtered with 1.3 mm pyrolytic graphite, 3.2 mm aluminium and 28 µm nickel, resulting in a beam with significant flux in the range of 18–35 keV and a peak energy of 25 keV. Spectral calculations were performed for the relevant undulator gaps using SPECTRA^57^. The effects of filters on these were calculated using attenuation coefficients from the Python library xraylib^58^. Samples were aligned for imaging under low-dose conditions (~10 minutes per sample) by temporarily setting the undulator gap to 10 mm^59^. Slits were used to restrict the beam just outside the FoV, making the effective beam area ~4.4 × 3.7 mm; this limited both sample exposure and the intensity of noise arising from scintillator defects.

Samples were placed on a HUBER 1002 manual goniometer (HUBER Diffraktionstechnik GmbH & Co. KG, Germany) (adjusted to make the spinal cords approximately vertical), mounted on perpendicular Newport MFA-PPD (Newport Corp., USA) linear stages, atop an Aerotech ABRT-260 (Aerotech Inc., USA) rotation stage. Projections were acquired at equally-spaced angles over 180° of continuous rotation (‘fly scan’), with an extra projection (not used for reconstructions) collected at 180° to check for possible sample deformation, bulk movements and beam damage relative to the first (0°) projection^59^. Projections were collected by a pco.edge 5.5 (PCO AG, Germany) detector (sCMOS sensor of 2560 × 2160 pixels) mounted on a visual light microscope of variable magnification. Magnification was controlled via rotation of a turret incorporating various scintillator-coupled objective lenses. A 2x objective, coupled to a 500 μm CdWO_4_ scintillator, mounted ahead of a 2x lens provided 4x total magnification, a FoV of 4.2 × 3.5 mm and an effective pixel size of 1.625 μm, as confirmed with a laminographic standard.

### Modular tomographic reconstruction with Savu

Data were reconstructed using the filtered back projection algorithm (FBP)^19^ running on a computer unified device architecture (CUDA) with the open source, modular pipeline Savu 2.2^59,60^. All images were first corrected for zingers - bright pixels resulting from the interaction of stray X-rays and cosmic rays with the sCMOS sensor^37^. Sample projections were then subject to flat- and dark-field correction, followed by a separate correction for radial distortion^61^, and another to suppress ring artefacts^32^. For the latter, distortion coefficients were calculated from visible light images of a Thorlabs Multi-Frequency Grid Distortion Target (Thorlabs Inc., USA). The distortion centre varied with rotations of the microscope turret and so was determined computationally by screening values which minimised differences between the first and last image of each scan (after the latter had been horizontally flipped and aligned to the former with the ImageJ *Template Matching and Slice Alignment* plugin^62^). For phase retrieval, a Paganin filter^63^ module was employed prior to reconstruction, with X-ray energy 25 keV, effective pixel size 1.6125 μm, propagation distance 160 mm and 𝛿/β ratios as per Fig. 4.

### Single-scan iterative downsampling to determine optimal projection number

The optimal projection number was determined with a single scan, iterative downsampling method. An ‘over-projected’ fly scan of 24,001 projections (step size 0.0225°, 80 ms exposure) was collected, then downsampled to evenly-spaced subsets of 12,000, 8,000, 6,000, 4,000, 3,000, 2,000, 1,500, 1,000, 750, 500, 375, 250 and 125 projections. Tomograms were reconstructed from the full 24,000 projection set and these subsets. The 80 ms exposure time was chosen to give a maximum of approximately 85% saturation (2^16^ counts) in flat-field images.

Image quality of subset tomograms was assessed by measuring peak signal-to-noise ratio (PSNR) and root mean square (RMS) contrast. Using the PSNR ImageJ plugin^64^, PSNR was computed by measuring the difference between a ‘true’ image (*t(x, y)*; the 24,000 projection tomogram) and ‘noisy’ image (*n(x, y)*; the downsampled tomogram) relative to the maximum signal in the ‘true’ image (Equation 2). RMS contrast was calculated on individually normalised tomogram slices cropped to a rectangular ROI around the sample, according to the equation of Peli^65^ (Equation 3). In these calculations a ROI was used (p(x,y)) rather than the entire image because this prevented background signal from dominating data.

2 $${\rm{PSNR}}=10\cdot {\mathrm{log}}_{10}[\frac{{\rm{\max }}\,{(t(x,y))}^{2}}{{\sum }_{0}^{{n}_{x}-1}{\sum }_{0}^{{n}_{y}-1}{[t(x,y)-n(x,y)]}^{2}}]$$

Peak signal to noise ratio (PSNR) for comparing noise between two images.

3 $$\begin{array}{c}{\rm{RMS}}={[\frac{1}{{n}_{x}{n}_{y}-1}\sum _{0}^{{n}_{x}-1}\sum _{0}^{{n}_{y}-1}{([p(x,y)-\bar{p})}^{2}]}^{1/2}\\ \bar{p}=\frac{1}{{n}_{x}{n}_{y}}\,\sum _{0}^{{n}_{x}-1}\sum _{0}^{{n}_{y}-1}p(x,\,y)\end{array}$$

Root mean square contrast (RMS) for calculating contrast within an image.

### Image processing, segmentation and analysis

To reduce file sizes, 32-bit depth tomograms were reduced to 16-bit and cropped to the region of interest using ImageJ (NIH, USA)^66^. Unfiltered data are presented throughout, except in Fig. 6 where minimum intensity projections of 2 collapsed tomogram slices are presented to emphasise vasculature and in Fig. 8, where a 2 × 2 × 2 3D median filter was used to reduce salt and pepper noise. Tomograms were matched to histology images by re-slicing and thickness adjusting in IMOD^67^. Spinal cord, white & grey matter and vasculature were segmented with the SuRVoS Workbench^38,39^. 3D renders were made in Avizo 9.4 (FEI Systems, Inc., USA) at I13-2^68^. All summary graphs were made using Prism 7.0a (GraphPad Software Inc., USA).

## Electronic supplementary material

Supplementary Information

Supplementary Video 1

Supplementary Video 2
