# Supplementary material for: Optimising complementary soft tissue synchrotron X-ray microtomography for reversibly-stained central nervous system samples
Source: Sci Rep. 2018 Aug 13;8:12017. doi: 10.1038/s41598-018-30520-8 (PMC6089931; doi:10.1038/s41598-018-30520-8)

Supplementary Information for

Optimising complementary soft tissue synchrotron X-ray microtomography for reversibly-stained central nervous system samples

Merrick C. Strotton[1,+], Andrew J. Bodey[2,+], Kazimir Wanelik[2], Michele C. Darrow[3], Esau Medina[1], Carl Hobbs[1], Christoph Rau[2], Elizabeth J. Bradbury[1,*].

[1]King's College London, Wolfson Centre for Age Related Diseases, Institute of Psychiatry, Psychology & Neuroscience, Guy's Campus, London Bridge, London, SE1 1UL, U.K.

[2]Diamond-Manchester Imaging Branchline I13-2, Diamond Light Source, Oxfordshire, OX11 0DE, UK.

[3]Beamline B24, Diamond Light Source, Oxfordshire, OX11 0DE, UK.

[*]Correspondence should be addressed to EJB (email: elizabeth.bradbury@kcl.ac.uk)

[+]Contributed equally to this work

Supplementary Legends

Supplementary Figure 1. 25 % Lugol's Iodine (LI) spinal cord histology and spinal cord tissue dimension changes induced by LI staining and paraffin wax embedding. 16 μm thick sections collected from **(A)** unstained cervical level spinal cord and **(B)** 25 % LI stained cervical level spinal cord were mounted in an aqueous embedding media to show tissue contrast (rather than normal xylene clearing and DePeX mounting which makes tissue transparent) and imaged under a bright field microscope. This demonstrated undetectable sample deformation by 25 % LI, but contrast changes within tissue. Tissue dimension changes of two PFA-fixed rat spinal cords divided into 4 roughly equal segments ~20 mm long, were assessed following either 25 % LI stain and paraffin embedding, or no stain and paraffin embedding. **(C)** Length and **(D)** width shrinkage of 10-15 % were noted in both LI and unstained segments by the final embedding stage relative to post-fixation dimensions. Segment **(E)** mass and **(F)** volume changes reveal **(G)** tissue density (mass/volume) increases of ~50 % following both procedures. Box and whisker plots show Min to Max of 4 segments for each condition. **(H)** All LI stained (brown) and unstained (white/clear) tissue segments used for dimension assessment photographed after the final Xylene clearing step. Matched LI and unstained tissues show similar proportions, with both treatments leading to some cord curling in the upper thoracic segment.

Supplementary Figure 2. Zinger artefact removal. Linear streaks in tomographic slices result from stray X-rays or cosmic rays directly striking the sCMOS detector. Though subtle, thresholding of difference maps calculated from tomographic slices derived from images **(A)** not processed for zinger removal with those **(B)** processed for zinger removal reveals **(C)** ~20 zingers per tomographic slice. Zingers can be identified at higher magnification in inset views of **(A', A'')** unprocessed tomograms relative to **(B', B'')** zinger-removed insets when guided by **(C', C'')** difference map insets. **(A***, yellow arrows**)** At high magnification, zingers can be more easily seen, but are **(B*)** no longer present in the zinger corrected images.

Supplementary Video 1. Transverse fly-through of the spinal cord volume (green) segmented from surrounding wax embedding media using the SuRVoS workbench (Figure 7.I).

Supplementary Video 2. Transverse fly-through of the spinal cord white and gray matter which has been hierarchically segmented within the spinal cord volume (Supplementary Video 1) using the SuRVoS Workbench (Figure 7.J-L).

Supplementary Figure S1

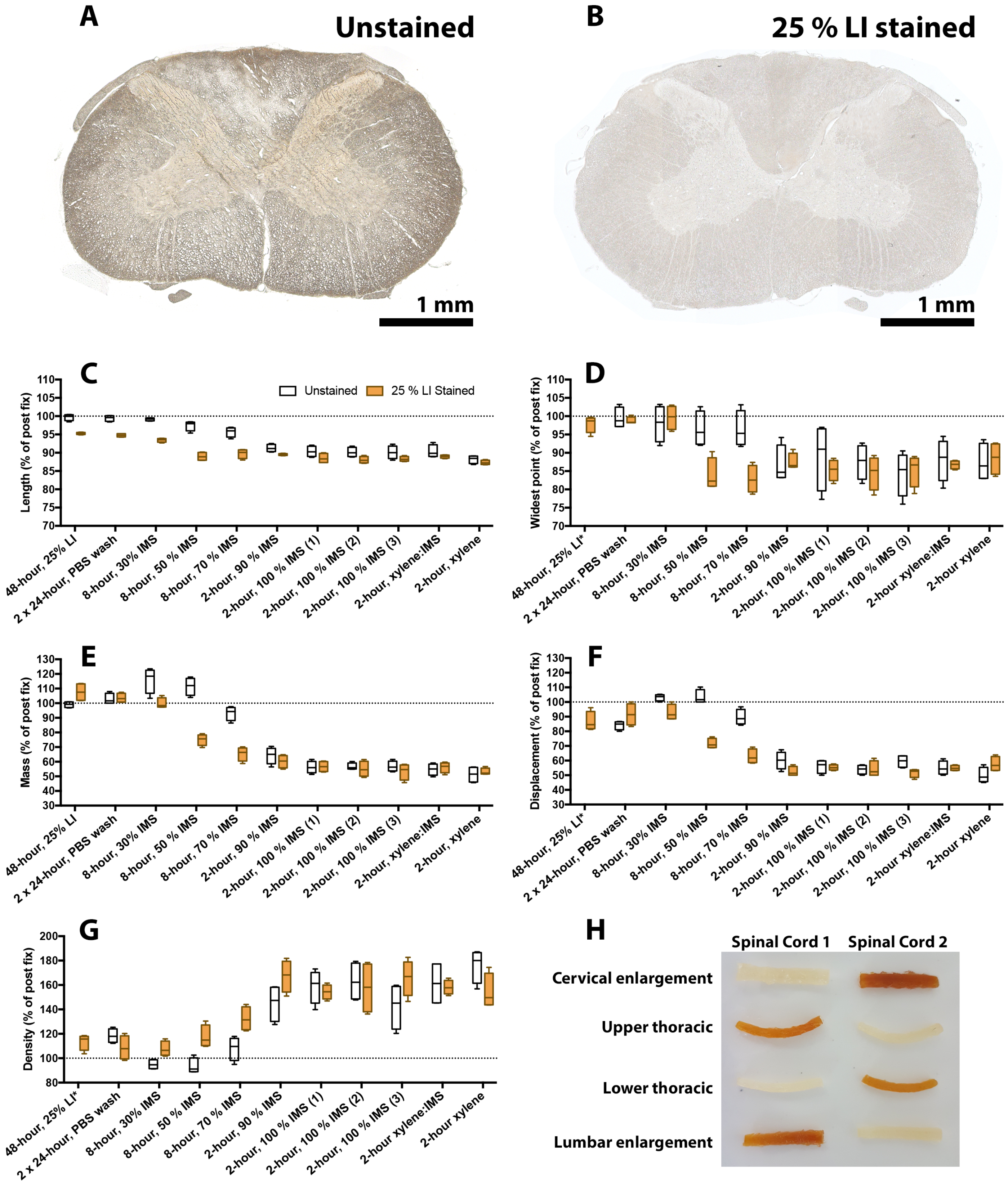

Supplementary Figure S2

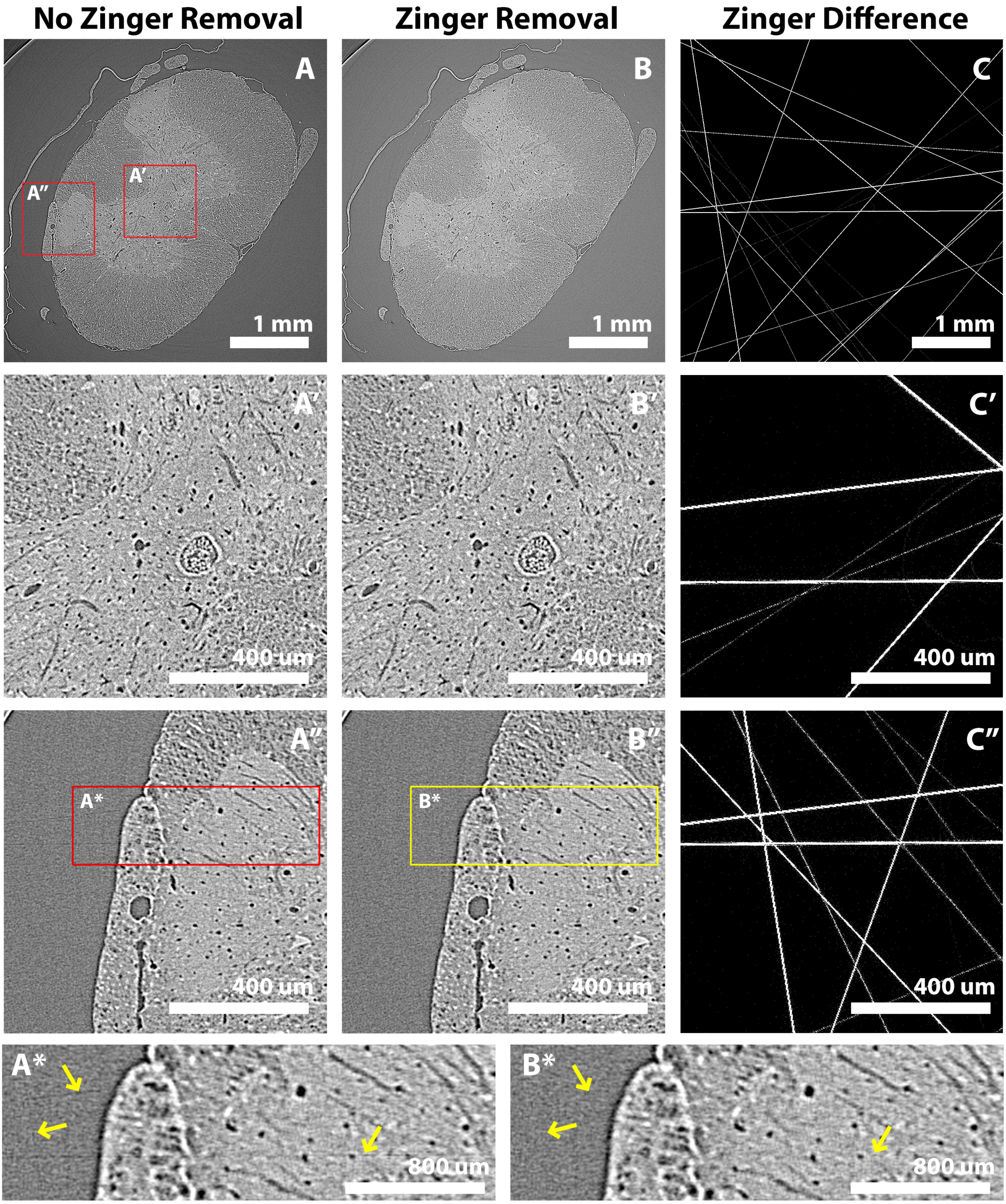

Supplement: Supplementary file 1 — Supplementary Information [file 41598_2018_30520_MOESM1_ESM.pdf]
